# A novel roseobacter phage possesses features of podoviruses, siphoviruses, prophages and gene transfer agents

**DOI:** 10.1038/srep30372

**Published:** 2016-07-27

**Authors:** Yuanchao Zhan, Sijun Huang, Sonja Voget, Meinhard Simon, Feng Chen

**Affiliations:** 1 Institution of Marine and Environmental Technology, University of Maryland Center for Environmental Science, USA; 2 CAS Key Laboratory of Tropical Marine Bio-resources and Ecology, South China Sea Institute of Oceanology, Chinese Academy of Sciences, Guangzhou, China; 3 Institute for Microbiology and Genetics. University of Göttingen, Germany; 4 Institute for Chemistry and Biology of the Marine Environment, University of Oldenburg, Germany

## Abstract

Bacteria in the Roseobacter lineage have been studied extensively due to their significant biogeochemical roles in the marine ecosystem. However, our knowledge on bacteriophage which infects the Roseobacter clade is still very limited. Here, we report a new bacteriophage, phage DSS3Φ8, which infects marine roseobacter *Ruegeria pomeroyi* DSS-3. DSS3Φ8 is a lytic siphovirus. Genomic analysis showed that DSS3Φ8 is most closely related to a group of siphoviruses, CbK-like phages, which infect freshwater bacterium *Caulobacter crescentus*. DSS3Φ8 contains a smaller capsid and has a reduced genome size (146 kb) compared to the CbK-like phages (205–279 kb). DSS3Φ8 contains the DNA polymerase gene which is closely related to T7-like podoviruses. DSS3Φ8 also contains the integrase and repressor genes, indicating its potential to involve in lysogenic cycle. In addition, four GTA (gene transfer agent) genes were identified in the DSS3Φ8 genome. Genomic analysis suggests that DSS3Φ8 is a highly mosaic phage that inherits the genetic features from siphoviruses, podoviruses, prophages and GTAs. This is the first report of CbK-like phages infecting marine bacteria. We believe phage isolation is still a powerful tool that can lead to discovery of new phages and help interpret the overwhelming unknown sequences in the viral metagenomics.

Bacteria in the Roseobacter clade are abundant and widely distributed in the marine environment^1^,^2^. The broad distribution of the Roseobacter clade is in nature largely attributed to their diverse metabolic capabilities^3^,^4^,^5^,^6^. Currently, more than 140 genomes of different roseobacter species have been sequenced, and analyses of representative genome sequences have generated new insight into ecological adaptation and evolution of the Roseobacter clade^7^.

Compared to the knowledge we gained on the Roseobacter clade, much less is known about bacteriophages which infect marine roseobacters (roseophage). Marine viruses, as the most abundant biological entities in the biosphere, play important roles in shaping host population structures, mediating genetic exchange between hosts, and modulating trophic transfer in marine food webs^8^,^9^,^10^,^11^. Viral metagenomic studies have greatly expanded our knowledge on viral diversity in nature^12^,^13^,^14^, but the interpretation of data is often hampered due to the limitation of our knowledge on isolated viruses. Recent isolations of phages infecting two abundant marine bacteria are the great examples for linking the unknown phages in the metagenomic database with known phages^15^,^16^. In addition, for the isolated phages, a wealth of biological information such as morphology, lytic activity, host specificity, genomics, and evolution can be retrieved.

Currently, only a handful of phages which infect roseobacters have been isolated and characterized. Roseophage SIO1 was the first described phage which infects *Roseobacter SIO67*^17^, and similar SIO1-like phages were found at the same site after 10 years^18^. Two N4-like phages infecting two different roseobacter strains (*Silicibacter pomeroyi* DSS-3 and *Sulfitobacter sp.* EE-36) were discovered, and they represent the first report of N4-like phages infecting marine bacteria^19^. Recently, several N4-like phages were isolated from different strains of the Roseobacter clade^20^,^21^,^22^, suggesting that N4-like phages may be a common type of phage infecting roseobacters. One siphovirus, phage RDJLΦ1, which infects *Roseobacter denitrificans* OCh114 was isolated from the South China Sea^23^. Most recently, two temperate podoviruses infecting *Sulfitobacter sp.* strain 2047 were also described^24^. These two temperate phages are almost identical at nucleotide level and were isolated from a mesocosm study in Raunefjorden.

The Roseobacter clade also contains inducible prophages. Prophages have been induced from two roseobacter strains, *Roseobacter* sp. TM1040^25^ and *Roseovarius nubinhibens* ISM^26^. Some bacterial strains (i.e. TM1040) may contain multiple prophages in their genomes^27^. Another phage-like structure, the gene transfer agent (GTA), has been found in nearly all the roseobacter genomes^7^,^28^,^29^. GTA is a phage-like entity that transfers random pieces of genome from donor cells to recipient cells^30^. Interestingly, roseophage RDJLΦ1 contains four GTA-like genes in its genome^31^. The Roseobacter clade are a group of highly diverse and adaptable bacteria. It is believed that phages (both lytic and temperate), prophages and GTA are the driving force for genomic diversification of the Roseobacter clade.

In this study, we report a new phage, phage DSS3Φ8, which infects a marine roseobacter, *R. pomeroyi* DSS-3. Phage DSS3Φ8 is a highly mosaic phage because it contains genetic elements of podoviruses, siphoviruses, prophages, and GTAs. To our knowledge, no phage similar to the very unusual DSS3Φ8 has been found in marine bacteria prior to this study.

## Results and Discussion

### Morphology and biological features

**Figure 1:**
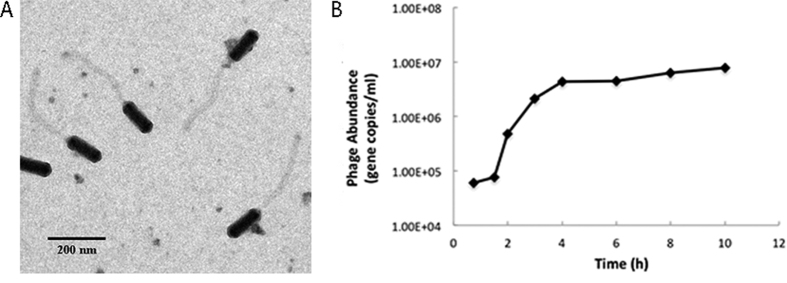
(**A**) Transmission electron microscopy image of roseophage DSS3Φ8. Scale bar = 200 nm. (**B**) One-step growth curve of roseophage DSS3Φ8.

Roseophage DSS3Φ8 was isolated from Baltimore Inner Harbor, Baltimore, USA in January 2012. DSS3Φ8 has a prolate cylindrical head and a long, flexible, non-contractile tail. The capsid size of DSS3Φ8 is ca. 130 nm long and 40 nm wide, and the tail length is ca. 300 nm (Fig. 1A). Based on its morphology, DSS3Φ8 is a member of B3 morphotype of *Siphoviridae*, which comprises approximately 1% of all known phages^32^. Genome sequence showed that DSS3Φ8 belongs to the phiCbK genus under *Siphoviridae*, which contains five B3 morphotype bacteriophages infecting the freshwater bacterium *Caulobacter crescentus*^33^. Phage CbK was first isolated in 1970, and has been an important genetic and cytological tool for studying cell cycle regulation, because it specifically infects “swarmer” host cells^34^. Roseophage DSS3Φ8 is much smaller than the five CbK-like phages in terms of capsid size and tail length.

The cross infectivity test showed that DSS3Φ8 did not infect the other four marine roseobacter strains, even though *Silicibacter* sp. TM1040 is in the same genus with DSS3. The latent period of DSSΦ8 was about 2 hours, and it reached the growth plateau in 6 hours. The burst size of DSSΦ8 is ca. 120 (Fig. 1B).

### General genome features

DSS3Φ8 is a circular, double-stranded DNA virus. The genome size of DSS3Φ8 is 146,135 bp. In general, the genome of DSS3Φ8 is homologous to that of the CbK-like phages (see the later section), but the genome size of DSS3Φ8 is much smaller compared to the known CbK-like phages (205–279 kb)^33^. The G + C content of the phage genome is 56%, lower than that of the CbK-like phages (62–66%). The G + C content is 64 and 67% for *R. pomeroyi* DSS3 and *Caulobacter crescentus*, respectively.

**Figure 2:**
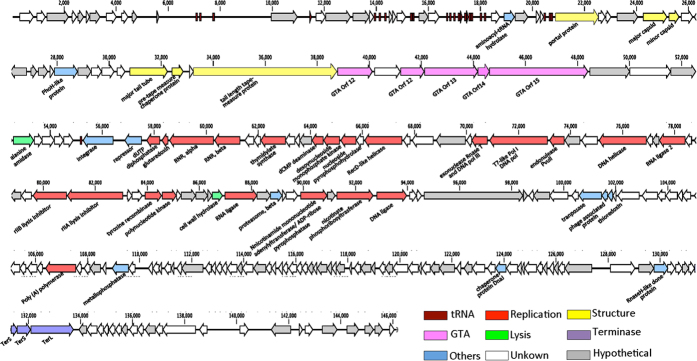
Genome map of roseophage DSS3Φ8. Gene features (unknown, hypothetical, tRNA genes, GTA and others) and genome modules (structure, lysis and DNA replication) are color-coded according to the legend below the figure.

A total of 229 predicted open reading frames (ORFs) was identified in the DSS3Φ8 genome, of which 106 ORFs had recognizable homologues in GenBank (Fig. 2). Among the recognizable proteins, only 50 have described function, consistent with the idea that marine siphoviruses encode proteins that are under-represented in the database^35^.

DSS3Φ8 contains 24 tRNA genes coding 14 different amino acids. Most of tRNA genes found in phage DSS3Φ8 overlapped with those found in the host *R. pomeroyi* DSS3. The parallel possession of tRNA genes between DSS3Φ8 and host DSS3 is considered as a result of co-evolution of phage and host^36^,^37^. On the other hand, two tRNAs, tRNA^Lys/CTT^ and tRNA^Gln/CTG^. were only found in DSS3Φ8. The anticodon CTT and CTG account for more than 90% codon usage for Lys and Gln respectively for phages, however, these anticodon are the rarest codons in the host^38^. This finding supported previous findings that phages tend to carry tRNAs corresponding to codons that are used frequently by the phage genes while rare in the host genome^39^. Also, it is possible that tRNA genes present in phages facilitate the expression of the late phage gene encoding structural proteins as proposed from cyanophage Syn9^40^.

### Homology between DSS3Φ8 and CbK-like phages

**Figure 3:**
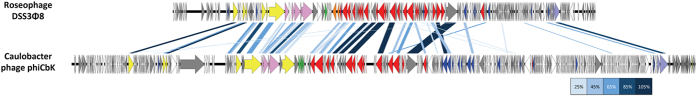
Genome wide comparison between roseophage DSS3Φ8 and *Caulobacter* phage phiCbK. White arrows represent ORFs for which no putative function can be attributed. Yellow, green, red and purple arrows represent structure, lysis, replication and packaging ORFs, respectively. Pink arrows stand for GTA structure, while orange arrows represent prophage-related ORFs. Grey color stands for hypothetical proteins. Related genes of these two phages are connected by blue shading. The color box corresponds to different amino acid identities.

**Figure 4:**
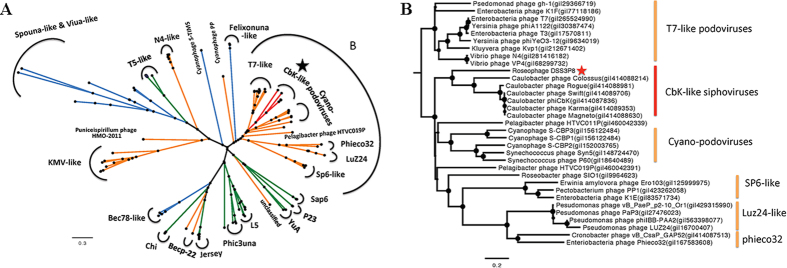
(**A**) The maximum likelihood phylogenetic tree of DNA polymerase I of bacteriophages. Orange, blue and green colors represent *Podoviridae, Myoviridae* and *Siphoviridae*, respectively. Red color represents the CbK-like phages. Bootstrap = 500. (**B**) Zoom in of region B in Fig. 4A.

Genome wide comparison between DSS3Φ8 and the CbK-like phages reveals that they all share similar modules (Fig. 3). Of the106 characterized ORFs with homologs in the DSS3Φ8 genome, 24 were related to the CbK-like phages, including the genes involved in DNA metabolism, replication, structure and genes with unknown function (Supplementary Table S1). The phylogenetic analyses based on the DNA polymerase gene (Fig. 4), major capsid gene (Supplementary Fig. S1) and terminase large subunit gene (Supplementary Fig. S2) all supported that DSS3Φ8 is more closely related to the CbK-like phages than to other phages. Also DSS3Φ8 forms a deep branch of its own among the CbK-like phages.

Although siphoviruses are usually highly mosaic in terms of their genomic evolution, siphoviruses with prolate capsid appear to maintain a conserved genomic architecture among them even though their genome sizes vary dramatically. For example, phage S-CBS2^41^, a siphovirus with prolate capsid, infecting a marine *Synechococcus* strain, exhibits a genomic arrangement similar to P-SS2^35^, a siphovirus with prolate head, infecting a marine *Prochlorococcus* strain. The genome size of phage S-CBS2, however, is only two thirds of that of phage P-SS2^41^. In our case, DSS3Φ8 infecting a marine alphaproteobacterium, *R. pomeroyi* DSS3, shared the highest genome homology with six siphoviruses CbK-like phages infecting a freshwater alphaproteobacterium, *Caulobacter crescentus,* despite that the genome size of DSS3Φ8 is about two-third of the genome sizes of CbK-like phages. The high genomic homology between DSS3Φ8 and CbK-like phages is remarkable because they infect bacteria inhabiting two different environments (marine vs. freshwater). A recent survey of the global oceans showed that siphoviruses with prolate capsids can make up 48% of siphoviruses observed in the marine environment^14^, suggesting that the genetic diversity of marine siphoviruses with prolate capsids remains largely unexplored.

### Difference between DSS3Φ8 and CbK-like phages

Despite the conserved genome arrangement between DSS3Φ8 and the CbK-like phages, an obvious genome reduction of DSS3Φ8 was observed. DSS3Φ8 contains 229 ORFs, while CbK-like phages have 318 to 448 ORFs^33^. The majority of missing genes are those without any matches in the NCBI database. The structure genes of DSS3Φ8 are much smaller than those of phiCbK, especially for the tail related genes. For instance, the length of pre-tape measure gene of DSS3Φ8 is half of phiCbK. The reduction of structure genes in DSS3Φ8 is consistent with the smaller head and shorter tail of DSS3Φ8 in comparison to the CbK-like phages. It appears that the loss of genes and the reduction of gene size both contribute to the reduction of the DSS3Φ8 genome.

For CbK-like phages, a lysis gene cassette coding for endolysin, holin and spanin was found between structure and replication gene cassettes. However, at the same region, DSS3Φ8 only contains one conseved lysis gene - peptidoglycan amidohydrolase (Fig. 3). On the other hand, DSS3Φ8 contains two hypothetical genes upstream to the endolysin gene, which we predict are involved in the lysis of the host cell based on their location.

### Replication System

DSS3Φ8 contains a conserved replication system, which includes genes coding for DNA polymerase, helicase, ligase, recombinase and nucleotide metabolism (Supplementary Table S1). DSS3Φ8 and the CbK-like phages are siphoviruses, but they likely hijacked the DNA polymerase gene from T7-like podoviruses. Such a genetic recombination between siphoviruses and podoviruses was first reported in the CbK like phages^33^. Phylogenetic analysis based on *polA* showed that DSS3Φ8 and the CbK-like phages form their own cluster within the *Podoviridae* clade, and closed related with T7-like *pol*A cluster (Fig. 4).

T7-like podoviruses typically contain a simple and conserved replication system, which includes the genes coding for DNA polymerase, helicase-primase, a single-stranded DNA binding protein, and thioredoxin^42^. DSS3Φ8 and the CbK-like phages possess the helicase gene, but lack the primase gene and the single strand binding protein gene. Possessing the thioredoxin gene may help proliferating phage DSS3Φ8 in marine environments since thioredoxin is known to promote DNA replication rate of T7-like DNA polymerase gene^43^. The thioredoxin gene has been found in many podoviruses infecting marine bacteria^44^,^45^.

### Prophage

DSS3Φ8 encodes an integrase and transcriptional regulator between the structure and replication modules, implying the lysogenic potential of this phage. The closest match (E-value < 2.5E-51, 32% identity) with the DSS3Φ8 integrase gene is in roseobacter *Ruegeria* sp. 6PALISEP08, which is 99% identical to the host strain *R. pomeroyi* DSS-3 based on the 16S rRNA sequences. The transcriptional regulator gene contains Cro/C1-type HTH and peptisase s24 domains, therefore was identified as a repressor. The genome of *R. pomeroyi* DSS-3 does not contain a prophage^38^. We searched for the site-specific attachment sites (*att*P and *att*B) in the genomes of phage DSS3Φ8 and host DSS-3, and found one possible attachment site, a 63bp sequence near the tRNA upstream of the integrase gene, which is 81% identical to the tRNA-Met4 gene in the host genome. Within the 63 bp region, a 18 bp segment was 100% identical between the phage and host. The *att*P-*att*B sites have been found in many bacteriophages including siphoviruses infecting marine picocyanobacteria^35^,^41^, and have been used to link phages with their potential hosts^46^. Because of the conserved nature of tRNAs, they can become the integration sites for temperate phages^47^. Whether this 63 bp region is the potential integration site for DSS3Φ8 remains to be tested.

### The GTA genes

Several hallmark genes of DSS3Φ8 are homologous to genes 12, 13, 14 and 15 in RcGTA, the gene transfer agent found in *Rhodobacter capsulatus*. These four GTA related genes have been found in the CbK-like phages^33^, phage ΦJL001 infecting marine *Rhizobiales str.* JL001^48^, and roseophage RDJLΦ1 infecting *Roseobacter denitrificans* Och114^31^. It is interesting that DSS3Φ8 carries two copies and larger size of RcGTA gene 12, which separated by an unknown protein.

**Figure 5:**
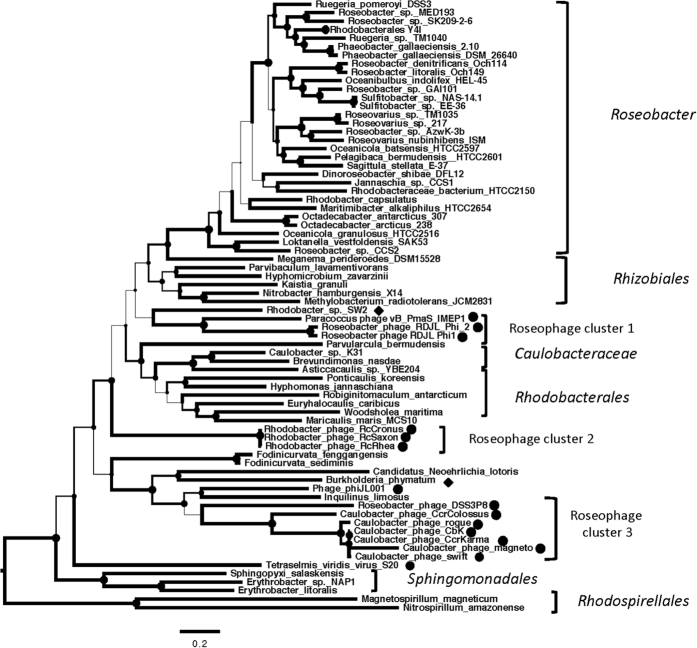
The maximum likelihood phylogenetic tree of concatenated protein sequences of RcGTA-like genes 12–15 from bacteriophages and bacteria. The circle represents the phage-derived four GTA genes, while the diamond indicates the GTA gene cluster came from the bacteria. Bootstrap = 500. The width of the branch corresponds to the bootstrap value.

GTA is a well-preserved genetic structure in alpha-proteobacteria, especially in the Roseobacter lineage. A phylogenetic analysis based on concatenated translated sequences of these four GTA genes showed that all members of roseobacters fell into their own clades (Fig. 5), which are consistent with the phylogeny based on their whole genome sequences^29^. Interestingly, phage-derived four GTA genes belonged to three different clades, which were not closely related to those of their potential hosts. This result suggests a host-independent evolution of phage-encoded GTA genes. Despite the fact that DSS3Φ8 and the CbK-like phages infect different bacterial species, the GTA gene phylogeny clusters these phages together, suggesting that DSS3Φ8 and the CbK-like phages acquired these four GTA genes from a comment ancestor (Fig. 5).

It is noteworthy that these four GTA genes are not found in several podoviruses including SIO-like phages and N4-like phages that infect different marine roseobacter strains^18^,^19^. It is possible that these four GTA genes are only common in siphoviruses infecting different roseobacter strains including phages RDJLΦ1, and DSS3Φ8. These four GTA genes were also found in a few unpublished siphoviruses infecting roseobacters (e.g. KT253150, NC_026608 and KT266805). The finding of roseophages carrying the four GTA genes indicates that there is a long and tangled evolutionary relationship between GTAs, phages and even prophages^50^. Isolation and genome sequencing of more roseophages will provide new insight into the evolutionary relationship between roseophages, GTAs and the Roseobacter clade.

### Environmental distribution of DSS3Φ8

**Figure 6:**
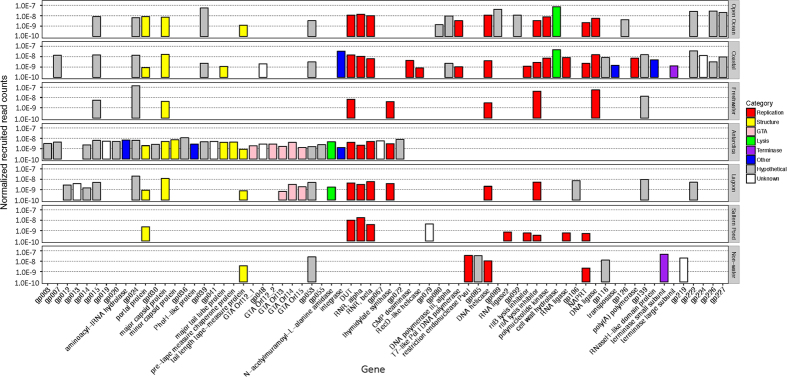
Rates of occurrences of DSS3Φ8 genes in various metagenomic databases. The colors of the bars indicate the categories of genes. The absolute counts of retrieved reads were normalized against the data sizes of metagenomes and the average gene size.

Metagenomic recruitments show that DSS3Φ8 reads were detected at the rates ranging from 10^−10^ to 10^−7^ per base pair in the metagenomic databases. The DSS3Φ8 homologs can be found in diverse habitats, ranging from freshwater to open ocean (Fig. 6). The highest recruitment rate came from the samples from Scripps Pier of Pacific Ocean Virome, where many SIO-like roseophages were isolated^17^,^18^. DSS3Φ8 was present in Antarctica, especially Organic Lake and Ace Lake, where abundant N4-like phages (Roseo N4-like phages) were observed^21^,^49^. DSS3Φ8 homologs were also recruited from other environments, such as chimney biofilm, stromatolite and whalebone. It appears that DSS3phi8-like phages are widespread in aquatic and marine environments.

## Conclusion

Lytic phage DSS3Φ8 is a new member of marine roseobacter phages, and represents the first CbK-like phage found in the marine environment. The genome sequence of DSS3Φ8 differs largely from all the known roseophages. DSS3Φ8 contains many features related to *Podoviridae, Siphoviridae*, prophage and GTA. Acquisition of T7-like DNA pol gene provides an efficient DNA replication mechanism to phage DSS3Φ8. On the other hand, carrying the integrase and repressor genes implies the lysogenic potential of DSS3Φ8. With its highly mosaic genome, DSS3Φ8 could serve as an ideal phage system to study the genomic evolution of phages and how the acquisition of different genetic elements (i.e. *pol, int*, or GTA genes) affect the phenotypic characteristics of phages.

## Methods and Materials

### Isolation of phage

Host stain *R. pomeroyi* DSS-3 was grown in ½ YTSS medium (4 g yeast extract, 2.5 g trypone and 20 g Crystal Sea per liter) at 28 °C. Water samples, collected from Baltimore Inner Harbor Pier V in January 2012, were filtered through 0.22 μm polycarbonate membrane filters (Millipore, Bedford, MA, USA). Filtrate of 10 ml was added into 50 ml of exponentially growing bacterial cultures and incubated for two days. The DSS-3 culture was filtered through 0.22 μm polycarbonate membrane filter (Millipore, Bedford, MA, USA) to remove bacteria. 100 μl of this cell-free lysate was added to 500 μl of exponentially growing DSS-3 culture, and plated using plaque assay. Phage isolates were purified three times by plaque assay.

### Transmission electron microscopy (TEM)

For TEM image, one drop of purified phage lysate was adsorbed to the 200-mesh Formvar/carbon-coated copper grid and stained with 1% phosphotungstic acid (PTA) for one minute^51^. Samples were examined with a JEOL JEM2100F transmission electron microscope, at the University of Oldenburg.

### Cross infection

Cross-infectivity of roseophage DSS3Φ8 was tested against other five marine roseobacter stains, including *Roseovarius nubinhibens* ISM, *Silicibacter* sp. TM1040, *Sulfitobacter* sp. EE-36, *Dinoroseobacter shibae* DFL-12 and *Roseobacter denitrificans* OCh 114. For each roseobacter strain, 500 μl of exponentially growing cells was added to 5 ml top agar and poured on the plates. After the agar was solidified, one drop of purified phage lysate was spotted onto each plate, and incubated for 2–3 days at 28 °C^18^. The formation of plaques was examined for possible cross-infectivity.

### One-step growth curve

One-step growth curve was determined using a method described elsewhere^52^. Generally, exponentially growing cultures of *R. pomeroyi* DSS-3 were incubated on ice for 20 min to synchronize the growth of host. After the DSS-3 culture was recovered to room temperature, roseophage DSS3Φ8 was added into the culture at a multiplicity of infection (moi) of 0.5. After 20 min attachment, cells were pelleted, re-suspended and diluted 100 times to avoid possible secondary infection. An aliquot of the cell suspension was collected at different time points between 30 min and 12 h.

The number of phages was enumerated by using qPCR^53^,^54^,^55^. Collected samples were filtered through 0.22 μm polycarbonate membrane filters (Millipore, Bedford, MA, USA) and DNA was extracted by WIZARD PCR Preps DNA Purification System (Promega Corporation, WI, USA). A set of PCR primers was designed based on the sequence of the phage DNA polymerase (Forward: ATGCTGCTCCGAACGTATCT, Reverse: ACTCGCCCTTCTTTTCCTTC). qPCR was conducted by APPLIED BIOSYSTEMS, 7500 Fast Real-time PCR system (ThermoFisher Scientific, Newyork, USA). qPCR reactions were performed in a 25 μl volume with 12.5 μl PERFECTA SYBR Green SuperMix (Quanta Biosciences, Gaithersburg, MD, USA), 400 nM forward and reverse primers and 5 μl of template. The amplification program was set as: 95 °C for 10 min, 40 cycle of 95 °C for 30 s, 60 °C for 30 s, 72 °C for 30 s. Fluorescence measurements were conducted at 60 °C of each cycle. Standards were developed using DNA extracted from phage lysate with known titer and 10-fold serial dilutions of the DNA were used in the reactions. Standard curves were determined as the correlation between the log of gene copy numbers and the Ct (R^2^ = 0.99).

### DNA extraction

One liter phage lysate was treated with DNase and RNase at a final concentration of 2 μg/ml (for both enzymes) at 4 °C to remove free DNA and RNA. Phage particles in the supernatant were precipitated with polyethylene glycol 8000 (final 10% w/v), and centrifuged in an OPTIPREP Density Gradient Medium for 12 h at 41,000 g. The visible viral band was extracted and dialyzed against TM buffer overnight at 4 °C. The purified phage particles were treated with a combination of sodium dodecyl sulfate (SDS, final concentration 1% w/v), proteinase K (final concentration 30 μg/ml) and EDTA (final concentration 5 μm) at 55 °C for 3 h. Phage DNA was then extracted using phenol/chloroform/isoamyl alcohol (25:24:1).

### Sequencing and annotation

The genome of roseophage DSS3Φ8 was sequenced by Illumina GAIIx sequencer. A 112 bp paired-end run resulted in 157.500 reads. The high quality reads were assembled by MIRA assembler, resulting in three contigs (139,090 bp, 2,108 bp and 4,767 bp in size). Primer walking with the Sanger sequencing method was followed to close the phage genome. Sanger sequencing was performed using ABI 3100 genetic analyzer (PE Applied Biosystems, Foster City, CA, USA) at the Institute of Marine and Environmental Technology, University of Maryland Center for Environmental Science. The circular feature of DSS3Φ8 was evident as we closed the genome sequences by primer walking from opposite directions.

The open reading frames (ORFs) were predicted using GeneMarkS^56^ and tRNAscan-SE^57^ was used to identify tRNA sequence. Translated ORFs were compared with known protein sequences using BLASTP (E-value < 10-5 and identity >30%). The complete genome sequence was submitted to GenBank with the accession number of KT870145.

Sequence alignment and phylogenetic analysis for *pol*A, major capsid protein, terminase large subunit and concatenated four GTA genes were performed using the CLC Genomic 7 program. A maximum-likelihood method was used to construct the phylogenetic tree, with bootstrap value of 500.

### Metagenomic recruitment

To explore the geographic distribution of DSS3Φ8, the phage genes were used as queries to search against all the available metagenomic databases in iMicrobe Database (http://data.imicrobe.us/) by June 2015. A reciprocal Blast analysis was conducted. Each of the recruited metagenomic reads was compared against the NCBI RefSeq database, which contains all complete viral and bacterial genomes by June 2015. The metagenomic reads were considered as DSS3Φ8 origin only if the DSS3Φ8 genes were the best hits. The relative abundance of each gene in every metagenomic database was calculated following the methods described elsewhere^15^. The count for each recruited read was divided by the number of total reads in the database, and further divided by the size of the gene product. The metagenomic databases were then categorized by their metadata. Samples were scaled using the mean of all samples.

## Additional Information

**How to cite this article**: Zhan, Y. *et al*. A novel roseobacter phage possesses features of podoviruses, siphoviruses, prophages and gene transfer agents. *Sci. Rep.* **6**, 30372; doi: 10.1038/srep30372 (2016).

## Supplementary Material

Supplementary Information
