# Supplementary material for: A novel roseobacter phage possesses features of podoviruses, siphoviruses, prophages and gene transfer agents
Source: Sci Rep. 2016 Jul 27;6:30372. doi: 10.1038/srep30372 (PMC4961962; doi:10.1038/srep30372)
Supplement: Supplementary Information [file srep30372-s1.pdf]

SUPPLEMENTARY FILES

A novel *Roseobacter* phage possesses features of podoviruses, siphoviruses, prophages and gene transfer agents

Yuanchao Zhan[1], Sijun Huang[2], Sonja Voget[3], Meinhard Simon[4], Feng Chen[1,*]

1. Institution of Marine and Environmental Technology, University of Maryland Center for Environmental Science, USA
2. CAS Key Laboratory of Tropical Marine Bio-resources and Ecology, South China Sea Institute of Oceanology, Chinese Academy of Sciences, Guangzhou, China
3. Institute for Microbiology and Genetics. University of Göttingen, Germany
4. Institute for Chemistry and Biology of the Marine Environment, University of Oldenburg, Germany

* Correspondence: chenf@umces.edu

Keywords: Roseobacter, *Ruegeria pomeroyi,* phage, CbK-like phage, GTA

Running title: A novel roseophage

Fig. S1. The maximum likelihood phylogenetic tree of major capsid protein of bacteriophages. CbK-like phages, available roseophages, typical marine bacteriophages and Enterobacter phages were selected as references. Bootstrap = 500.

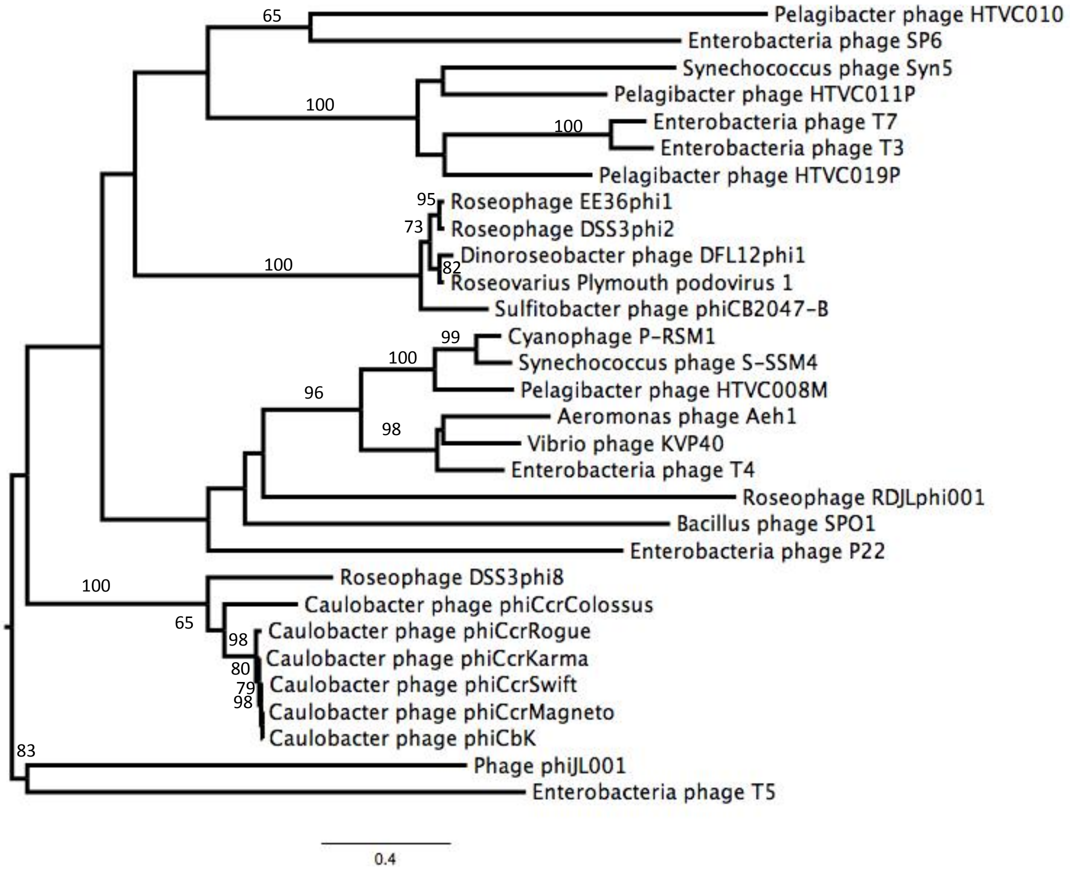

Fig. S2. The maximum likelihood phylogenetic tree of terminase large subunit of bacteriophages. CbK-like phages, available roseophages, typical marine bacteriophages and Enterobacter phages were selected as references. Bootstrap = 500.

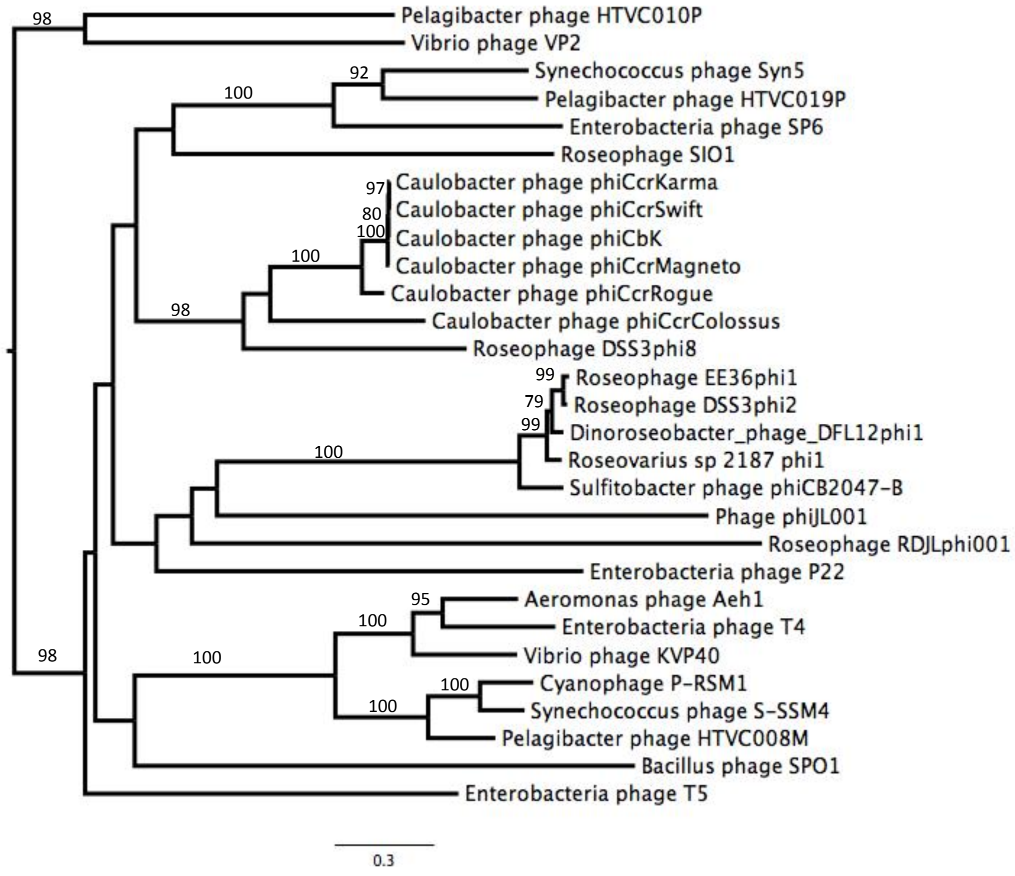

Table S1. Description of characterized ORFs of phage DSS3Φ8 and its kinship with the CbK-like phages

Orf Name	Start	End	Length of amino acid	Strain	Description	Homolog to CbK-like phage*
DSS3P8_gp023	18900	19313	138	+	putative aminoacyl-tRNA hydrolase	+
DSS3P8_gp027	20891	22531	547	+	putative portal protein	+
DSS3P8_gp030	23245	24051	269	+	hypothetical protein	+
DSS3P8_gp031	24249	25175	309	+	putative major capsid protein	+
DSS3P8_gp032	25238	25642	135	+	putative minor capsid protein	+
DSS3P8_gp034	26831	26205	209	-	transcriptional regulator	
DSS3P8_gp035	26995	27222	76	+	hypothetical protein	+
DSS3P8_gp038	27889	28794	302	+	PhoH-like protein	+
DSS3P8_gp039	28794	29312	173	+	hypothetical protein	+
DSS3P8_gp043	30791	32251	487	+	major tail tube protein	+
DSS3P8_gp044	32406	32855	150	+	pre-tape measure chaperone protein	+
DSS3P8_gp046	33256	38733	1826	+	phage tail length tape-measure protein	+
DSS3P8_gp047	38775	40121	449	+	GTA Orf12	+
DSS3P8_gp049	41202	42116	305	+	GTA Orf12	
DSS3P8_gp050	42113	44173	687	+	GTA Orf13	
DSS3P8_gp051	44173	44622	150	+	GTA Orf14 (NlpC/P60 family cell wall peptidase)	+
DSS3P8_gp052	44612	48403	1264	+	GTA Orf15	
DSS3P8_gp056	52579	53370	264	+	N-acetylmuramoyl-L-alanine amidase	
DSS3P8_gp063	58220	57705	172	-	Deoxyuridine 5'-triphosphate nucleotidohydrolase	
DSS3P8_gp064	58526	58290	79	-	putative glutaredoxin protein	
DSS3P8_gp065	60286	58583	568	-	Ribonucleotide reductase, alpha subunit	
DSS3P8_gp066	61294	60314	327	-	Ribonucleotide reductase, beta subunit	
DSS3P8_gp069	63042	62089	318	-	thymidylate synthase	

DSS3P8_gp073	64502	64056	149	-	CMP deaminase	
DSS3P8_gp074	65130	64504	209	-	deoxynucleotide monophosphate kinase	
DSS3P8_gp075	65763	65164	200	-	nucleotide pyrophosphohydrolase	
DSS3P8_gp077	67519	66083	479	-	putative RecD-like helicase	+
DSS3P8_gp080	69964	68831	378	-	hypothetical protein	+
DSS3P8_gp083	73119	70885	745	-	putative T7-like Pol I DNA polymerase	+
DSS3P8_gp084	73756	73187	190	-	type II restriction endonuclease PvuII	
DSS3P8_gp087	76912	75092	607	-	DNA helicase	+
DSS3P8_gp090	78404	77403	334	-	RnlB RNA ligase2	
DSS3P8_gp094	80893	79604	430	-	Phage rIIB lysis inhibitor	
DSS3P8_gp095	83066	80919	716	-	Phage rIIA lysis inhibitor	
DSS3P8_gp098	83904	84551	216	+	putative tyrosine recombinase	+
DSS3P8_gp099	84567	85118	184	+	polynucleotide kinase	
DSS3P8_gp104	86488	86895	136	+	cell wall hydrolase	
DSS3P8_gp105	86975	88219	415	+	RNA ligase	
DSS3P8_gp107	88728	89186	153	+	proteasome subunit beta	
DSS3P8_gp110	89882	90913	344	+	bifunctional nicotinamide mononucleotide adenylyltransferase/ ADP-ribose pyrophosphatase	
DSS3P8_gp112	91272	92681	470	+	nicotinate phosphoribosyltransferase	
DSS3P8_gp113	92816	93973	386	+	DNA ligase	
DSS3P8_gp117	98440	98613	58	+	hypothetical protein	+
DSS3P8_gp119	99460	98840	207	-	Type I secretion protein	
DSS3P8_gp122	101454	100585	290	-	transposase	
DSS3P8_gp129	104376	103924	151	-	phage prohead protease	
DSS3P8_gp131	104890	104570	107	-	thioredoxin	
DSS3P8_gp136	107556	106381	392	-	poly(A) polymerase	
DSS3P8_gp139	108496	108095	134	-	hypothetical protein	+

DSS3P8_gp141	109574	108939	212	-	putative metallophosphatase	
DSS3P8_gp181	121519	119435	695	-	hypothetical protein	+
DSS3P8_gp182	122100	121516	195	-	hypothetical protein	+
DSS3P8_gp187	124081	123674	136	-	putative chaperone protein DnaJ	
DSS3P8_gp199	129170	129763	198	+	hypothetical protein	+
DSS3P8_gp200	129760	130302	181	+	putative RNaseH-like domain protein	+
DSS3P8_gp205	131324	131575	84	+	terminase small subunit	
DSS3P8_gp206	131562	132086	175	+	terminase small subunit	
DSS3P8_gp207	132083	133732	550	+	putative terminase large subunit	+

*: + in the column indicates that the top tBLASTx hit of respective gene is one of CbK-like phages.
